# Multiple hats, one truth: Lived experience as knowledge

**DOI:** 10.1371/journal.pmen.0000676

**Published:** 2026-11-04

**Authors:** Stéfanie Fréel, Sasha Hajj Assaf, Adrián Pérez Ara, Peter Varnum, Naeem Dalal, Emmanuelle Verkest

**Affiliations:** 1 Global Mental Health Solutions, Geneva, Switzerland; 2 Amsterdam UMC, Department of Psychiatry, location University of Amsterdam, Amsterdam, The Netherlands; 3 Justice for Mental Health Association, Beirut, Lebanon; 4 Orygen Global, Caracas, Venezuela; 5 Zambia National Public Health Institute, Lusaka, Zambia; 6 Lille Neuroscience and Cognition Centre (U-1172), Univ Lille, INSERM, CHU Lille, Lille, France; 7 Centre National de Ressources et Résilience pour les psychotraumatismes (CN2R Lille - Paris), Lille, France; PLOS: Public Library of Science, UNITED KINGDOM OF GREAT BRITAIN AND NORTHERN IRELAND

## Abstract

The global mental health field increasingly recognizes the importance of involving people with lived experience of mental ill-health in research, policy, services, and systems. However, recognition and representation have not consistently translated into meaningful authority. The current essay examines the conceptual and structural questions underlying this gap: what constitutes experiential knowledge; how lived experience is transformed into expertise; how experiential, clinical, academic, and policy knowledge interact; and how institutions can distinguish genuine co-production from symbolic participation or incognito tokenism. Drawing on participatory traditions within and beyond mental health and community-led practice, the essay argues that experiential knowledge is neither a substitute for professional expertise nor subordinate to it. Instead, these forms of knowledge should operate reciprocally, with epistemic authority shifting according to whose knowledge is most relevant to the question at hand. At the same time, the authors distinguish this fluid exchange of epistemic authority from the more entrenched institutional power governing resources, leadership, and decision-making. The essay examines how power, institutional readiness, context, crisis, and intersectional diversity shape whether participation becomes structural or merely symbolic. A case study from Nunavut, Canada, illustrates both the consequences of externally imposed systems and the possibilities created when Indigenous knowledge and authority determine terms of engagement. Practical recommendations for moving from consultation toward co-governance are put forward to support decision-makers within global mental health seeking to operationalize meaningful lived-experience engagement rather than simply endorse it.

## Introduction

The global mental health field increasingly recognizes that people with lived experience (PWLE) of mental ill-health must play a substantive role in shaping research, policy, services, and systems [1]. The experiential knowledge they bring is distinct from, and complementary to, clinical, academic, and policy expertise [2]. It can illuminate how systems are encountered in everyday life, where barriers arise, and what meaningful support looks like from the perspective of those most directly affected [3–5].

Yet recognition does not necessarily translate into authority. Experiential knowledge remains structurally fragile: PWLE are invited into institutions, but often on terms defined by those institutions [6,7]. As professionals who have worked in global mental health for decades, and as PWLE ourselves, we examine several unresolved questions: how lived experience is defined and by whom [8]; whether having lived experience automatically confers expertise; how experiential knowledge relates to other forms of knowledge; and how institutions can distinguish meaningful co-production from symbolic participation [9].

We approach these questions through our own experiences as global mental health professionals who have grappled with multiple identities and competencies throughout our careers. We have ourselves encountered programs and activities that instrumentalize lived experience: the use of personal stories or dual‑role professionals to demonstrate inclusion while leaving governance, resources, and decision‑making power largely unchanged [4,5]. We argue instead for a reciprocal model in which experiential and professional knowledge challenge, legitimize, and transform one another.

To structure our discussion, we adopted a fishbone as a conceptual heuristic to map multi‑level barriers and facilitators to meaningful participation of PWLE. This conceptual lens helped us organize themes into broad domains and derive the guiding framework for this essay. No primary data were collected, and the fishbone was not used as a formal root‑cause analysis but as a scaffold for the narrative argument.

### The lived experience movement in context: Historical participatory traditions

For much of modern mental health history, professional, clinical, and academic knowledge dominated formal knowledge production, with services delivered *to* people rather than designed *with* them. The growth of consumer, service-user, survivor, ex-patient, Mad Pride, psychosocial disability, Hearing Voices, neurodiversity, and broader disability-rights movements challenged this hierarchy. Their advocacy, including the principle “Nothing About Us Without Us,” helped shift PWLE from being treated primarily as patients, research participants, or beneficiaries toward being recognized as partners and knowledge-holders [10–13].

The recognition of experiential knowledge in mental health also builds on participatory traditions that emerged during the HIV/AIDS epidemic when effective treatments were unavailable and institutional responses were often inadequate. People living with HIV/AIDS generated and mobilized knowledge grounded in their own lived experience, becoming indispensable partners in research rather than passive recipients of care. Their activism profoundly reshaped biomedical research, clinical trials, and health policy, establishing a model of knowledge co-production that continues to influence participatory approaches across health disciplines and actors [14–17]. Despite these advances, institutional adoption of meaningful engagement of PWLE in policies and programs designed to support them has been uneven, and formal inclusion can still reproduce the same hierarchies it claims to address.

### What is experiential knowledge?

To date, theoretical frameworks have defined experiential knowledge in terms of its content (e.g., [18,19]), nature (e.g., [20]), function (e.g., [21]), mobilization potential (e.g., [22]), anticipated outcomes (e.g., [23]), and relation to identity and socioeconomic status (e.g., [19,24,25]). While an in-depth discussion of the existing literature is beyond the scope of this essay, experiential knowledge represents the capacity to interpret, mobilize, and transform direct experience of mental ill-health, recovery, caregiving, or mental health systems into knowledge that can inform action [24,26,27]. It includes phenomenological insight, practical know-how, relational understanding, and a form of receptivity and attunement to the lived, embodied, and relational dimensions of mental ill-health and recovery, enabling the recognition and interpretation of experiences within their specific social and personal contexts [25].

Experiential knowledge moves beyond a clinical or academic *comprehension* of a system’s or service’s function and efficacy, to understanding how these are *experienced* from a human perspective [28]. It is contextual, subjective, emotional, tacit, and embedded not only in practice, but also in people's experiences of systems, services, and mental states [29–31]. Experiential knowledge develops through processes of meaning-making, articulation, and dialogue, through which lived experiences can be transformed into knowledge that is communicable and relevant within collaborative contexts [32]. It is therefore contextual, situated, and heterogeneous. This situatedness is also reflected in scholarship developed with PWLE across different social and cultural contexts, including participatory work with peer researchers in Ghana and Indonesia [4] and co-written scholarship in which experts by experience have contributed directly to the interpretation and articulation of experiential knowledge [5,33].

Experiential knowledge is neither a substitute for clinical or academic knowledge nor inherently superior to it. Rather, it offers a different vantage point. Like a tenant who understands what it feels like to live in a building which architects and engineers designed and constructed, experiential experts contribute knowledge inaccessible through professional perspectives alone. This knowledge may be individual or collective, is shaped by identity and context, and evolves over time. It reflects both an individual's experience and the broader realities of communities interacting with mental health systems [34]. Distinct individuals may experience the same mental health condition, service, or system differently because of their cultural background and identity, the sociocultural, politico-economic context they live in, and the resulting dynamics created from encounters with health and social care systems [27,34,35].

However, experiential knowledge does not only constitute knowledge derived from lived experience; it is also a legitimate form of epistemology. The epistemic validity and legitimacy of experiential knowledge stands in the notion that direct, lived experience represents a fundamentally unrivalled form of knowing and acquaintance with a particular phenomenon such as mental ill-health and/or recovery [36]. In fact, Borkman (1976) refers to experiential knowledge as constituting a primary source of truth with equivalent epistemological authority to professional knowledge [27]. In this view, experiential knowledge constitutes the wisdom and know-how derived from personal participation in a phenomenon [27], paired with the individual’s conviction surrounding its validity and authority, characterized as individual, pragmatic, holistic, and focused on action in the here and now [27,37].

The definition of what constitutes experiential knowledge has since been vastly debated, expanded on and diversified, encompassing the practical know-how, subjective understanding, tacit wisdom, as well as the embodied and relational dimensions of adversity, mental ill-health and recovery, shaped by interactions with mental health and/or traditional care systems and broader socio-cultural, historical and structural dynamics [25,38,39]. Experiential knowledge has further been defined as a composite of knowledge forms including propositional (knowing that), practical (knowing how) and acquaintance knowledge (derived from bodily experience), transforming over time [20,40]. More recent debates qualify experiential knowledge as representing knowledge both about the world and one’s capabilities within it, where epistemic patterns of lived experience encompass the individual’s experience of illness, the know-how derived from it, the knowledge developed at a collective level, and associated experiential expertise - the ability to not just translate one form of knowing to another but to effectively mobilize it, acknowledging the impact of context and ongoing power dynamics [37].

The authors of this article thus adopt the perspective that experiential knowledge should be viewed as a dynamic continuum between lay and biomedical knowledge, where experiential and scientific knowledge are not opposing but complementary in nature, continuously shaped by the realities of living with mental health challenges, the context(s) within which these occur and the intersecting systems which individuals interact with [19,37,41]. While we recognize and value the heterogeneity and plurality of knowledge types held by any given individual – as well as their ability to co-exist, the authors adopt the view that experiential knowledge can only become transformed into, and mobilized as, expertise once it is fully accepted, appropriated, and owned by the individual and integrated into their identity (see, e.g., [19,37,42,43]. In our view, this process is dynamic, multidirectional and continuously evolving, acting to both facilitate and maintain recovery by offering a palette of knowledge, wisdom, tools, and capabilities which the individual can draw upon in context to actively engage with, and shape, the systems surrounding them. As such, the meaning, interpretation, narrative and depth surrounding one’s lived experience is continuously transformed through interactions with oneself – including one’s beliefs, values and identities, ongoing experiences, encounters with the broader collective and the wider socio-political dynamics that contribute to shaping it (see, e.g., [44–46]).

### Why is experiential knowledge important?

At its most fundamental level, the inclusion of experiential knowledge in research and practice is central to addressing the needs – and respecting the choices of - persons living with mental ill-health within the contextual realities they live in [19,24,47]. It ensures that research, care and policy are driven by a plurality and diversity of voices, ultimately strengthening the relevance, acceptance and efficacy of mental health services, programs and systems and safeguarding their cultural competency [48]. By capturing everyday realities, unintended consequences, barriers, gaps, and facilitators experienced by PWLE, their caregivers and communities, experiential knowledge complements professional understanding [8,49]. Indeed, it contributes a distinct and applied form of expertise to strengthen research, policy, and practice [8,49]. Its value lies in examining the topic of concern through the perspective of those who have directly experienced it, thereby complementing and enriching other ways of knowing [8].

The significance of lived experience in mental health research and practice is, however, much broader, grounded in civil rights movements and social justice frameworks. Historically, movements led by PWLE of mental ill-health challenged paternalistic care models and questioned the exclusion of service users from research, policy, service planning and decision-making. These movements reframed lived experience not merely as a source of personal testimony, but as a legitimate form of knowledge and a basis for claims to autonomy, participation, and epistemic recognition [7,50]. In this way, experiential research and practice represent a paradigm shift, empowering persons living with mental health conditions - and their carers, to voice, choose and actively shape services and systems that work *with* and *for* them, not *on* them [51]. Experiential knowledge challenges conventional epistemic hierarchies by positioning lived experience as a legitimate form of expertise and fostering the co-production of knowledge between PWLE, researchers, and practitioners [7,27], contributing to more equitable and responsive mental health systems [51].

Achieving this requires a shift away from divisive, imbalanced top-down approaches grounded in an “us-vs-them” mentality, to an active, non-hierarchical collaboration grounded in humility, which actively addresses power dynamics and challenges preconceptions [48]. In this way, lived experience practice represents a *bridging* of parties traditionally involved in producing research, delivering care, and implementing policies, and those affected by them [15]. In this active and dynamic framework, various forms of knowledge – scientific, clinical, and experiential – are acknowledged as credible sources of “truth” and equally valued [27,52,53], promoting mutual learning and knowledge co-creation [54], and reducing stigma [55]. As such, lived experience research and practice not only promote the co-design and co-production of person-centered, rights-based policies and services, but directly foster empowerment and epistemic justice [54].

### Who qualifies as a person with lived experience?

In a strict view, a “PWLE” refers to a person who is a partner and collaborator – not merely a clinical patient – and can transform their lived experience into knowledge in practical ways to shape mental health research, policy or services. PWLE can develop this skill set either through formal training or acquired experience [20,56]. Importantly, the presence of lived experience does not necessarily imply that the person *wishes* to mobilize or share it in a particular context. In fact, even when a person chooses to draw upon their lived experience, this does not automatically equate to experiential knowledge being mobilized. The way in which lived experience is recognized, valued, and transformed into knowledge represents a dynamic process dependent on the person, context, and purpose of the involvement [27,32].

Naturally, lived experience expertise is diverse, including persons living with, or recovered from, a mental health condition, survivors or perpetrators (e.g., of sexual, gender, intimate partner, sectarian or political violence, human trafficking, genocides, armed conflict, terrorism, mass accidents), caregivers, relatives or next of kin [24]. Just as a plurality of knowledge forms co-exist *among* actors involved in lived experience research and practice, so too can these coincide *within* a given individual [44,45,57]. Lived experience experts may be mental health researchers, clinicians, policymakers, service implementers, or advocates, simultaneously holding various forms of knowledge [57].

Importantly, the dual roles which stakeholders bring to the table – i.e., individuals with combined lived experience of mental health challenges and professional expertise – can be seen as an advantage. Because many people who work in the field of global mental health have personal motivations for doing so, many who are researchers, service implementers, or clinicians also have lived experience. Instead of treating these categories as rigid and distinct, stakeholders should see the value these multiple forms of expertise can bring, for example in bridging existing institutional structures with independent or emerging people or groups with lived experience, or ensuring mental health research, practice and policy processes are grounded in reflexivity, relationality, and social accountability [54]. This liaising could come in the form of making connections to lesser known networks, groups or individuals; demystifying jargon; or formal or informal mentorship. Indeed, such activities are a form of redistributing power that will benefit the field at large.

While a diversity of knowledge sources may foster more fluid identity boundaries, encourage advocacy and allow for the cross-pollination of knowledge across multiple roles, it also holds risks [57–60]. These may include the intentional or unintended marginalization or exclusion of persons exclusively holding experiential knowledge from co-production and decision-making processes [6,7], the creation of power hierarchies amongst groups of PWLE [60], and the reinforcement of stereotypes, discriminatory practices, divisive structures and censorship around lived experience disclosures [57,59]. For this reason, governance mechanisms (see, e.g., [38,61,62]) should be designed for this type of dual-expertise, while simultaneously ensuring that lived experience is recognized as a category of expertise alongside other forms of knowledge. Having dual expertise does not mean one’s opinions are more valid; but rather that they are informed by a more nuanced understanding of the issue at hand where personal meaning and professional rigor are integrated and actively drawn upon [63–65].

Finally, experiential knowledge should not be treated as a homogeneous category [8,27]. People with lived experience are often expected to represent diverse diagnoses, identities, and intersecting social positions, though no two individuals experience the same condition or recovery in the same way [29,34]. Such an approach risks privileging certain voices while excluding others, particularly those with more complex support needs [8]. Meaningful participation therefore requires care about whose lived experience is being sought, whether it is representative, and how diverse expertise is brought together without reducing it to a single narrative or creating hierarchies amongst lived experience experts [8,27,34].

### How is experiential knowledge co-created and shared?

Experiential knowledge is therefore diverse and, at times, conflicting - a contradiction which should not be viewed as a limitation, but rather as one of its greatest strengths. Meaningful knowledge creation requires careful consideration of *whose* experiential knowledge is being sought, *why* it is being engaged, and *how* diverse voices are included in the process [49]. It necessitates a recognition of heterogeneity both within and across PWLE, while moving away from hierarchies and stereotypes that position one identity or form of knowledge as superior to another [8]. As such, meaningful knowledge creation is necessarily predicated upon conceptual humility: the recognition that no single discipline, profession, or individual holds a complete understanding of complex human experiences [66–69]. This requires acknowledging the limits of one's own knowledge, remaining open to revising existing assumptions, and recognizing that experiential knowledge can reveal dimensions of mental health research and service delivery that may remain inaccessible through traditional academic and clinical perspectives alone [48,70].

To achieve conceptual humility, it is crucial that all parties to lived experience research and practice are sensitized – ideally through formal training and capacity strengthening - to i) the importance of the role(s) they hold within a given context, ii) the type(s) of knowledge forms which are being mobilized, and the identity being held, by the individual provided the situational needs, and iii) associated risks of unintentionally leveraging a multiplicity of roles and knowledge to yield coercive power (see, e.g., [57,71]). Clarifying roles internally and externally can go a long way toward operationalizing safeguards in establishing supportive structures for collaboration. This process might include clarity on when to engage lived experience research and practice, developing a common charter outlining shared values and principles codeveloped and agreed on by all parties. It may also involve introducing individuals external to the process – such as facilitators or coordinators, who can identify and address power imbalances, facilitate contentious discussions, and help uphold the charter’s content and values.

However, these attempts cannot, by themselves, create the conditions for genuine knowledge sharing and power redistribution. Building meaningful partnerships requires an informed commitment from all parties — comparable to the principle of informed consent in healthcare — understood not simply as agreement to participate, but as a genuine willingness to enter a shared process of learning and transformation [48,71]. Achieving this requires the recognition that no singular individual or group possesses complete knowledge, and that meaningful collaboration depends on accepting one’s own limitations while trusting that others’ perspectives, experiences, and forms of expertise can address what one does not know [7,69,71]. Partnership is therefore not achieved by bringing together already complete forms of knowledge, but by creating the conditions in which knowledge can be challenged, expanded, and co-created.

### What does co-design and co-production mean within the context of experiential knowledge?

To fulfil their collaborative purpose, co-design and co-production mechanisms must recognize PWLE not merely as contributors to research and practice, but as active and equal partners in decision-making and knowledge production [72]. Meaningful co-design and co-production depend not on *whether* participation occurs but rather on *how* it occurs. It places responsibility not only on the willingness of PWLE to participate, but also on the appropriateness of the conditions within knowledge-production systems and the intention of all parties to be active participants and co-constructors of knowledge [72]. Successful collaboration requires genuine power-sharing, whereby experiential expertise is included at key decision points throughout the entire process, rather than being consulted after decisions have already been made or at a single, limited stage of the work [73]. This is the key difference between meaningful engagement and tokenism – where PWLE are consulted and engaged in performative and unequal ways.

The use of participatory approaches does not imply the *replacement* of scientific rigor with flexibility, but rather an attempt to *balance and strengthen* it. The meaningful co-design and co-production of knowledge also requires acknowledging - and working with - historical exclusion, trauma, and injustice, while intentionally creating space for voices traditionally marginalized within mental health research and practice, allowing experiential knowledge to guide inclusion [10,73]. Equally important is genuine humility and self-awareness among all collaborators, recognizing and addressing the assumptions and positions of power each individual consciously or unconsciously brings into the process, while remaining open to reflecting on and challenging these, to meet on common ground. When these conditions are met, co-design and co-production move participation beyond consultation and symbolic involvement toward person-centered, rights-based mutual ownership and accountability [72].

### Barriers to meaningful co-design and co-production

One of the most persistent barriers to meaningful participation is tokenism: PWLE are provided with a seat at the table but little authority over what happens at it [8–10]. Participation becomes symbolic, with experiential experts consulted while having limited influence over decisions, governance, funding, or knowledge production, often because power is unequally shared and experiential knowledge is still treated as secondary to professional expertise [9,72]. Tokenism can also hide in plain sight as “incognito tokenism,” where institutions satisfy participatory requirements by relying on professionals or academics with lived experience while overlooking independent experiential experts [8,10].

Such dynamics reflect epistemic injustice: experiential knowledge is acknowledged, but not yet equally valued across leadership, healthcare, policy, and research (see, e.g., [7,8,10,29]. Co-production can still reproduce existing hierarchies, expecting experiential knowledge to fit within pre-existing scientific frameworks [9]. Research conducted through the GOAL project on the Mental Health and Psychosocial Support (MHPSS) Taskforce in Lebanon [74] illustrates this: despite being nationally led, decision-making power remained concentrated among United Nations agencies and international organizations with funding and institutional credibility. Differences in seniority, language, meeting formats, and existing relationships with leadership influenced whose voices were heard, so that participation ranged from merely being informed to genuine partnership [73].

Meaningful participation also depends on whether organizations are ready to host it. Much of the literature focuses on whether PWLE are ready to participate, with little attention paid to institutional readiness to share power [72]. In practice, organizations often seek participation without the governance, resources, and supportive environments required to sustain it. Unclear roles, inaccessible processes, jargon, missing accommodations and lack of remuneration place the burden of inclusion on PWLE rather than on the systems that hold decision-making power [8,10,72]. Without adequate support, participation itself can carry real costs, including emotional burden, re-traumatization, and the strain of balancing engagement with health, employment, and everyday life demands [8,10,72].

### Conditions for meaningful participation of experiential knowledge

If tokenism gives people a seat without a say, the answer is not more seats but a real shift in who holds power. Destrooper (2015) calls this “reverse standard-setting,” where those most affected shape practice rather than receive it as passive participants [75]. Meaningful co-production means sharing real authority over governance, funding, and knowledge itself, and doing so from a project’s start, not at a final consultation once decisions are made [9]. This takes more than presence: it means being honest about who truly shaped a decision, and confronting the gatekeeping that confines experiential leaders to advisory roles rather than decision-making positions. Where power stays concentrated, more seats change nothing; where experiential knowledge becomes the ground for collective power, participation transforms. For example, Talcott (2014) record through their writings how women in Oaxaca, Mexico, turned their own experiences of violence and trauma into the basis for Indigenous-led organizing [76]. Either way, no amount of participation closes the gap between presence and power without redistributing it.

Power-sharing also centers on whose knowledge counts. Here authority is less a fixed possession than something that shifts as different expertise becomes relevant, so the aim is not to displace professional knowledge but to stop ranking experiential knowledge beneath it [32]. Recognition on paper is not the same as respect in practice: if experiential knowledge is welcomed in principle but still ranked below clinical or academic credentials, co-production rebuilds the hierarchy it claims to dismantle [77]. Treating it as equal expertise means letting communities define what good outcomes look like, paying experiential experts properly, opening leadership opportunities to them, and accepting knowledge and programs that do not fit pre-defined templates. The real question is not whether such programs satisfy outside norms, but whether the communities they serve find them valuable. Fig 1 provides a case example from Nunavut, Canada, illustrating both the consequences of externally imposed systems and the possibilities created when Indigenous knowledge and authority determine terms of engagement.

**Fig 1:**
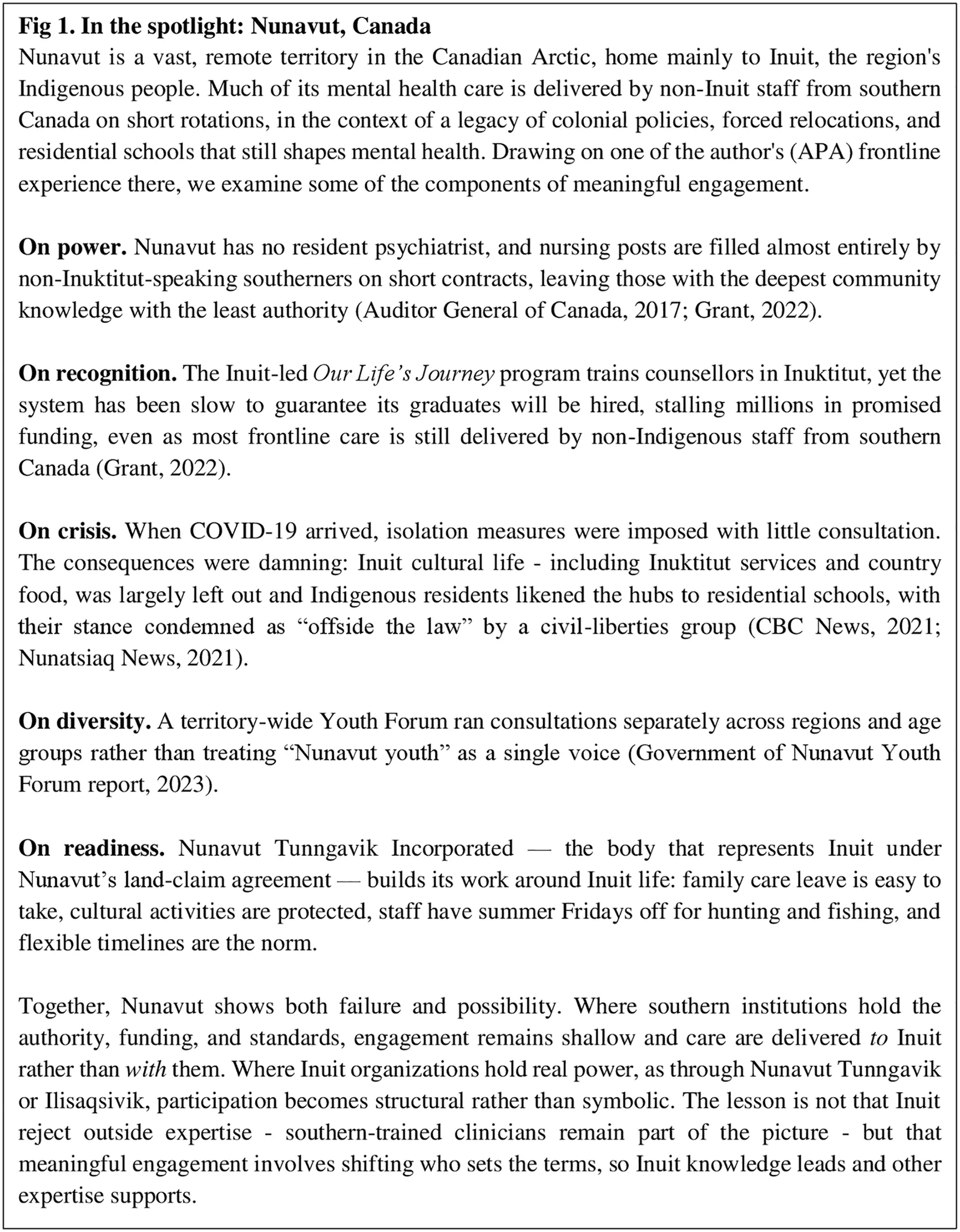
In the spotlight: Nunavut, Canada [79–81].

Shared power and recognition still need an organization ready to hold them. Too often the burden falls on PWLE while the institutions inviting them in remain unchanged [72]. Before seeking participation, organizations should put the basics in place: varied recruitment, clear roles, accessible meetings and plain language, capacity strengthening, fair pay, and protected time. These considerations should exist alongside real care for people, from cultural, psychological, and physical safety and trauma-informed practice to mentorship respecting fluctuations in mental health status [4,5]. Organizations can also hire people with lived experience into positions of power. Readiness like this cannot be improvised once a project is underway.

Even a ready organization is tested by its context, because participation never happens in a vacuum. Conflict, humanitarian emergencies, environmental and climate-related disasters, and structural inequality shape who takes part and whose knowledge counts as legitimate [72]. The danger is that crises become an excuse to set participation aside, so decisions are made for people rather than with them while the underlying structures stay intact, even though a crisis is often when those affected understand the stakes most clearly. As an example, in Kurdistan-Iraq, women who survived the 1988 Anfal genocide wanted to be remembered as strong survivors, yet a dominant narrative of victimhood came to define them and their recovery, overriding their own accounts [78]. Context-sensitive participation means adapting to culture and circumstance as they evolve, empowering affected communities, and securing a clear community mandate (i.e., an endorsement from the affected community and its leaders) before acting. Emergencies should test these commitments, not suspend them.

## Conclusion: The structural crisis of power in global mental health

The global mental health field has reached a decisive inflection point. The question of whether experiential knowledge matters is no longer contested; ethical, clinical, and empirical consensus affirms its value. The unresolved problem is structural: rhetorical recognition has far outpaced the redistribution of power [6].

Across research, policy, clinical care, and emergency response, the recurring failure is not exclusion but tokenistic presence without authority. This “incognito tokenism” allows institutions to satisfy participatory check‑boxes by leveraging dual‑role professionals while systematically withholding co‑leadership from independent experiential experts [73]. The result is symbolic inclusion without structural transformation [9]. Yet, with appropriate institutional incentives, training and supportive policies surrounding disclosure and support (e.g., CARRY practices [63]), dual-role professionals may be uniquely positioned to act as brokers, translators, or facilitators across a plurality of experiential and professional knowledge forms [38,63]. By navigating both personal and professional identities, these individuals may contribute to building bridges across distinct epistemic centers of authority, helping to surface the implicit hierarchies influencing power imbalances in perceived legitimacy of experiential knowledge [82]. In this way, dual-role professionals may act as conduits in legitimizing and centering the voices of PWLE and those often excluded from traditional knowledge systems in person-centered research, policy and care [63,83,84].

Meaningful participation cannot be reduced to whether PWLE are “in the room.” It requires intentionally reckoning with both structural power and epistemic dynamics. Often, discussions about power in participatory spaces evoke an adversarial, political narrative of “power-sharing” that can trigger unnecessary apprehension among researchers, clinicians or policy-makers, who may perceive it as a challenge to their professional roles.

In practice, however, collaborative research and care is characterized not by a struggle over fixed roles, but by the dynamic exchange of epistemic authority. Influence emerges from the complementary nature of distinct perspectives. While a lived-experience expert's knowledge might redirect a project by identifying a critical, overlooked dimension, a researcher’s methodological expertise simultaneously guides its appropriate execution [2]. In this view, authority naturally shifts to whoever holds the most relevant knowledge to the question at hand. This recognition moves us beyond defensive, adversarial dynamics toward a more nuanced, collaborative model oriented toward improving the quality of science.

Crucially, we must distinguish this fluid, contextual epistemic authority from the rigid institutional and political power governance, funding, and decision-making which remains embedded within organizational structures. The goal is not to replace professional with experiential authority, but to construct a reciprocal model where the two continuously legitimize, challenge, and transform one another.

Ultimately, the central crisis in meaningful engagement of PWLE within global mental health remains structural. Without redistributing institutional power, recognition remains rhetorical; but without protecting dynamic epistemic authority, collaboration remains frozen in apprehension. The way forward is to embed lived‑experience authority into systems’ architecture, ensuring co‑production is not symbolic but operational [8,9].

### Recommendations

In light of this essay, the authors put forward seven structural transformations required to move from consultation to co‑governance:

1. **Co‑governance, not consultation**. Embed lived‑experience authority into governance, funding, and research frameworks and structures from inception, with PWLE holding minimum voting equity thresholds across major decision points to ensure shared ownership.
2. **Participation as infrastructure**. Allocate a specific budget line to projects and initiatives, so as to guarantee fair remuneration, protected time, accommodations, and structured peer mentorship. Specific amounts should be aligned with recognised lived experience remuneration frameworks or principles, such as those articulated in the World Health Organization meaningful engagement framework [61] or the aves Mental Health framework [62]. This alignment allows for flexibility according to the scope, context, and level of involvement required. Inclusion must be resourced through non-divertible budget lines, not discretionary.
3. **Conceptual humility as institutional**. Implement institutional mechanisms predicating partnership models on conceptual humility, reciprocity and complementarity, acknowledging that no one source of knowledge holds more truth or authority than – nor displaces, another. Create dedicated dialogic spaces and institutional practices that promote open, candid and continuous exchanges to identify, acknowledge and address power dynamics and preconceptions, while mainstreaming participatory principles into internal institutional policies, governance models, and leadership training and capacity strengthening.
4. **Anti‑tokenism safeguards**. Implement explicit anti-tokenism gatekeeping mechanisms to safeguard the integrity of co-production. This may include differentiating internal vs. external roles, adopting co‑developed charters of values, and using independent facilitation to monitor and address power balances to prevent the displacement, or outvoting, of single‑role experiential experts.
5. **De-medicalize metrics**. Widen the epistemological boundaries of research, clinical and programmatic frameworks in global mental health to ensure success and clinical outcomes are measured and validated according to communities’ lived experiences, on their own terms. This may include creating monitoring, evaluation and learning mechanisms driven rather than informed by lived experience, and establishing transparent institutional pathways to list independent peer leaders as Co-Principal Investigators (Co-PIs), evaluators, and primary authors.
6. **Hold the line in times of crisis**. Participatory safeguards must not be suspended during acute public health emergencies, humanitarian crises, or socio-economic shocks. Health security architectures must resist defaulting to exclusionary, top-down command structures. Instead, community-led feedback loops must be integrated into standard operating procedures for emergency preparedness and response, for example by embedding a permanent “Community Advisory Node” directly into formal Incident Management Systems (IMS) and early warning systems.
7. **Intersectional representation**. Lived experience represents a heterogeneous, multi-layered data stream - resist the single‑representative trap. Use matrixed selection frameworks to track intersectional variables and ensure diversity across age, sex, gender, geography, socio‑economic status, diagnosis, and cultural background, while explicitly including carers as independent experts.

## References

[1] McGorry PD, Mei C, Dalal N, Alvarez-Jimenez M, Blakemore S-J, Browne V, et al. The lancet psychiatry commission on youth mental health. Lancet Psychiatry. 2024;11(9):731–74. doi: 10.1016/S2215-0366(24)00163-9 39147461

[2] Okoroji C, Mackay T, Robotham D, Beckford D, Pinfold V. Epistemic injustice and mental health research: A pragmatic approach to working with lived experience expertise. Front Psychiatry. 2023;14:1114725. doi: 10.3389/fpsyt.2023.1114725 37056406 PMC10086175

[3] Killackey E. Lived, loved, laboured, and learned: experience in youth mental health research. Lancet Psychiatry. 2023;10(12):916–8. doi: 10.1016/S2215-0366(23)00270-5 37625427

[4] Tizaa HL, Aboagye LAF, Drah EA, Ofosuware JS, Faustina E, Nurmalasari DE, et al. Lived experience in mental health research in Ghana and Indonesia: What have we learned?. PLOS Ment Health. 2025;2(6):e0000344. doi: 10.1371/journal.pmen.0000344 PMC12798261 41661873

[5] Sunkel C, Sartor C. Perspectives: Involving persons with lived experience of mental health conditions in service delivery, development and leadership. BJPsych Bull. 2022;46(3):160–4. doi: 10.1192/bjb.2021.51 33977895 PMC9346508

[6] Catala A. The dynamics of epistemic injustice: Situating epistemic power and agency. New York: Oxford University Press; 2025.

[7] Fricker M. Epistemic injustice: Power and the ethics of knowing. Online edn ed. Oxford Academic; 2007. doi: 10.1093/acprof:oso/9780198237907.001.0001

[8] Samra R. Conceptualising lived experience in mental health research: Problems, insights and implications. Wiley Online Library. 2025;47(e70039). doi: 10.1111/1467-9566.70039 PMC12043252 40305649

[9] Turnhout E, Metze T, Wyborn C, Klenk N, Louder E. The politics of co-production: participation, power, and transformation. Curr Opin Environ Sustain. 2020;42:15–21. doi: 10.1016/j.cosust.2019.11.009

[10] Taylor S. The wounded healer: The history and implications of lived experience in mental health care. Australas Psychiatry. 2025;33(3):339–41. doi: 10.1177/10398562241303267 39622620

[11] Redit C. Lived experience: shifting focus. Nat Ment Health. 2023;1:145. doi: doi.org/10.1038/s44220-023-00040-0

[12] Banfield M, Palmer VJ. Embedding lived experience in mental health research: what we need to pack (and unpack) for the future in mental health research and translation. BMJ Open. 2025;15(5):e098557. doi: 10.1136/bmjopen-2024-098557 40447441 PMC12128406

[13] Gaynor-Brook L. Towards mental health as a human right: The key role of lived experience. PLOS Med. 2023;20(10):e1004307. doi: 10.1371/journal.pmed.1004307 PMC10564147 37816029

[14] Epstein S. Impure science AIDS, activism, and the politics of knowledge. University of California Press; 1996. 11619509

[15] Callon M, Lascoumes P, Barthe Y. Acting in an uncertain world: An essay on technical democracy. Bijker W, Jones-Imhotep E, Slayton R, editors. Cambridge, MA, USA: MIT Press; 2011.

[16] Israel BA, Schulz AJ, Parker EA, Becker AB. Review of community-based research: Assessing partnership approaches to improve public health. Annu Rev Public Health. 1998;19:173–202. doi: 10.1146/annurev.publhealth.19.1.173 9611617

[17] Minkler M, Wallerstein N. Community based participatory research for health: Process to outcomes. San Francisco: Jossey-Bass; 2008.

[18] Dumez V, L’Espérance A. Beyond experiential knowledge: A classification of patient knowledge. Soc Theory Health. 2024;22(3):173–86. doi: 10.1057/s41285-024-00208-3

[19] Jovchelovitch S. Knowledge in context representations, community and culture. Routledge/Taylor & Francis Group; 2007.

[20] Caron-Flinterman JF, Broerse JEW, Bunders JFG. The experiential knowledge of patients: a new resource for biomedical research?. Soc Sci Med. 2005;60(11):2575–84. doi: 10.1016/j.socscimed.2004.11.023 15814182

[21] Abel EK, Browner CH. Selective compliance with biomedical authority and the uses of experiential knowledge. Pragmatic women and body politics. Cambridge University Press; 1998. 310–26.

[22] Gross O, Gagnayre R. Caractéristiques des savoirs des patients et liens avec leurs pouvoirs d’action: implication pour la formation médicale. Rev Fr Pédagogie Rech En Éducation. 2017;(201):201. doi: 10.4000/rfp.7266

[23] Jouet E, Flora LG, Las Vergnas O. Construction et reconnaissance des savoirs expérientiels des patients. Prat Form Rev Int Sci Hum Soc. 2010;2010:olivier_lv.

[24] Halloy A, Simon E, Hejoaka F. Defining patient’s experiential knowledge: Who, what and how patients know. A narrative critical review. Sociol Health Illn. 2023;45(2):405–22. doi: 10.1111/1467-9566.13588 36443986 PMC10100415

[25] Dings R, Strijbos DW. Being in a position to know: Attuned responsiveness as the hallmark of experiential knowledge and expertise in mental healthcare. Front Psychiatry. 2025;15:1490489. doi: 10.3389/fpsyt.2024.1490489 39872434 PMC11770679

[26] Castro EM, Van Regenmortel T, Sermeus W, Vanhaecht K. Patients’ experiential knowledge and expertise in health care: A hybrid concept analysis. Soc Theory Health. 2018;17(3):307–30. doi: 10.1057/s41285-018-0081-6

[27] Borkman T. Experiential knowledge: A new concept for the analysis of self-help groups. Soc Serv Rev. 1976;50(3):445–56. doi: 10.1086/643401

[28] Nagel T. What is it like to be a bat?. Philos Rev. 1974;83(4):435–50. doi: 10.2307/2183914

[29] Boardman FK. Knowledge is power? The role of experiential knowledge in genetically “risky” reproductive decisions. Sociol Health Illn. 2014;36(1):137–50. doi: 10.1111/1467-9566.12048 24111508

[30] Borgdorff H. The conflict of the faculties: Perspectives on artistic research and academia. Leiden University Press; 2012. doi: 10.26530/OAPEN_595042

[31] Polanyi M. The tacit dimension. Sen A, editor. Chicago, IL: University of Chicago Press; 2009.

[32] Verkest E, Borsellino J, Creupelandt C. Du savoir expérientiel au partenariat: l’alliance des savoirs. Inf Psychiatr. 2025;101(6):393–9. doi: 10.1684/ipe.2025.2898

[33] Fusar-Poli P, Estradé A, Stanghellini G, Venables J, Onwumere J, Messas G, et al. The lived experience of psychosis: A bottom-up review co-written by experts by experience and academics. World Psychiatry. 2022;21(2):168–88. doi: 10.1002/wps.20959 35524616 PMC9077608

[34] Berg BL. Qualitative research methods for the social sciences. Boston, Mass., Munich: Pearson; 2010. 432.

[35] da Cunha Lewin CC. What kind of lived experience knowledge is the right kind? A reflective commentary. Soc Psych Psychiatr Epidemiol. 2025;60(6):1511–4. doi: 10.1007/s00127-025-02823-5 39966166 PMC12162775

[36] Bergqvist A, Crepaz-Keay D, Wilde A. Introduction: What is the role of lived experience in research?. R Inst Philos Suppl. 2023;94:1–14. doi: 10.1017/S1358246123000292

[37] Halloy A, Simon E, Hejoaka F. Defining patient’s experiential knowledge: Who, what and how patients know. A narrative critical review. Sociol Health Illn. 2023;45(2):405–22. doi: 10.1111/1467-9566.13588 36443986 PMC10100415

[38] Festervoll A, Lara EI, Gavin C, Mercier N, Norton MJ, Speyer H. Five meaningful roles of engagement: Framework for lived experience roles in mental health research (FLER-R). PLOS Ment Health. 2026;3(8):e0000632. doi: 10.1371/journal.pmen.0000632 PMC13496407 42627835

[39] World Health Organization WHO. Transforming mental health through lived experience: roadmap for integrating lived and living experience practitioners into policy, services and community. 2025. https://www.who.int/europe/publications/i/item/WHO-EURO-2025-12307-52079-79927

[40] Gardien E. L’accompagnement et le soutien par les pairs. 2017. Accessed 2026 September 30.

[41] Prior L. Belief, knowledge and expertise: The emergence of the lay expert in medical sociology. Sociol Health Illn. 2003;25:41–57. doi: 10.1111/1467-9566.00339 14498929

[42] Blume S. In search of experiential knowledge. Innov Eur J Soc Sci Res. 2016;30(1):91–103. doi: 10.1080/13511610.2016.1210505

[43] Mazanderani F, Noorani T, Dudhwala F, Kamwendo ZT. Knowledge, evidence, expertise? The epistemics of experience in contemporary healthcare. Evidence & Policy. 2020;16(2):267–84. doi: 10.1332/174426420x15808912561112

[44] Näslund H. Collective deliberations and hearts on fire: Experiential knowledge among entrepreneurs and organisations in the mental health service user movement. Volunt Int J Volunt Nonprofit Organ. 2022;33(1):201–12. doi: 10.1007/s11266-020-00233-6

[45] Noorani T, Karlsson M, Borkman T. Deep experiential knowledge: Reflections from mutual aid groups for evidence-based practice. Evid Policy. 2019;15(2). doi: 10.1332/174426419x15468575283765

[46] Pols J, Hoogsteyns M. Shaping the subject of incontinence. Alter Eur J Disabil Res. 2016;10(1):40–53. doi: 10.1016/j.alter.2015.07.005

[47] Pols J. Knowing patients: Turning patient knowledge into science. Sci Technol Hum Values. 2014;39(1):73–97. doi: 10.1177/0162243913504306

[48] Israel B, Eng E, Schulz A, Parker E. Methods for community-based participatory research for health. 2nd ed. 2012.

[49] Nimkulrat N, Groth C, Tomico O, Valle-Noronha J. Knowing together – experiential knowledge and collaboration. CoDesign. 2020;16(4):267–73. doi: 10.1080/15710882.2020.1823995

[50] Beresford P. Service users’ knowledges and social work theory: Conflict or collaboration?. British J Social Work. 2000;30(4):489–503. doi: 10.1093/bjsw/30.4.489

[51] Faulkner A, Basset T. A helping hand: taking peer support into the 21st century. Mental Health and Social Inclusion. 2012;16(1):41–7. doi: 10.1108/20428301211205892

[52] Gieryn TF. Cultural boundaries of science: credibility on the line. Chicago, IL: University of Chicago Press;1999.

[53] März J, Arui AK, Rutjens BT. Understanding experiential and subjective truth: Development and validation of the ‘Youniversalism’ scale. Personal Individ Differ. 2026;258:113820. doi: 10.1016/j.paid.2026.113820

[54] Johner Z, Klenk N, Cvitanovic C, Borie M. How can we better account for experiential knowledge in co-production frameworks?. Environ Sci Policy. 2025;171:104183. doi: 10.1016/j.envsci.2025.104183

[55] Kohrt B, Jordans M, Turner E. Collaboration with people with lived experience of mental illness to reduce stigma and improve primary care services: A pilot cluster randomized clinical trial. JAMA Network Open. 2021;4(11):e2131475. doi: 10.1001/jamanetworkopen.2021.31475 PMC8567115 34730821

[56] Gross L. L’engagement des patients au service du système de santé. Paris: Doin Editions; 2017.

[57] Hawke LD, Sheikhan NY, Jones N, Slade M, Soklaridis S, Wells S, et al. Embedding lived experience into mental health academic research organizations: Critical reflections. Health Expect. 2022;25(5):2299–305. doi: 10.1111/hex.13586 35999670 PMC9615091

[58] Lupton D. The commodification of patient opinion: The digital patient experience economy in the age of big data. Sociol Health Illn. 2014;36(6):856–69. doi: 10.1111/1467-9566.12109 24443847

[59] Varghese FP, Boyd JE. Clinicians with lived experience of mental illness: Introduction to a special section on prosumers, in honor of the late Dr. Fredrick J. Frese III. Psychol Serv. 2022;19(1):1–5. doi: 10.1037/ser0000613 35175087

[60] Mazanderani F, Noorani T, Dudhwala F, Kamwendo ZT. Knowledge, evidence, expertise? The epistemics of experience in contemporary healthcare. Evid Policy. 2020;16(2):267–84. doi: 10.1332/174426420x15808912561112

[61] World Health Organization WHO. WHO framework for meaningful engagement of people living with noncommunicable diseases, and mental health and neurological conditions. 2023. https://www.who.int/publications/i/item/9789240073074

[62] From recognition to realisation: equal pay for work of equal value lived experience expertise in global mental health collaborating with health. Accessed 2023 October 1. https://www.aves-mh.org/files/aves%20MH%20EbE%20Renumeration%20Framework%20-%20LE%20Inclusion%20Assessment%20Tools.pdf

[63] Hunt DF. Holding duality in mental health research and practice: Towards the meaningful integration of dual-positioned researchers. Critical Public Health. 2026;36(1). doi: 10.1080/09581596.2026.2619251

[64] Clark M. Co-production in mental health care. Ment Health Rev J. 2015;20(4):213–9. doi: 10.1108/MHRJ-10-2015-0030

[65] Kidd SA, Kral MJ. Practicing participatory action research. J Couns Psychol. 2005;52(2):187–95. doi: 10.1037/0022-0167.52.2.187

[66] Staiti A. The pedagogic impulse of Husserl’s ways into transcendental phenomenology. Grad Fac Philos J. 2012;33(1):39–56. doi: 10.5840/gfpj20123313

[67] Solomon M. Scientific rationality and human reasoning. Philos Sci. 1992;59(3):439–55. doi: 10.1086/289680

[68] Hoekstra R, Vazire S. Aspiring to greater intellectual humility in science. Nat Hum Behav. 2021;5(12):1602–7. doi: 10.1038/s41562-021-01203-8 34711978

[69] Longino H. Science as social knowledge. Princeton University Press; 1990.

[70] Moran D. Introduction to phenomenology. London and New York: Routledge; 2000.

[71] Cornish F, Breton N, Moreno-Tabarez U, Delgado J, Rua M, de-Graft Aikins A, et al. Participatory action research. Nat Rev Methods Primers. 2023;3(1). doi: 10.1038/s43586-023-00214-1

[72] Lokot M, Zreik T. Research co-production within humanitarian health: Reflections on our practice. Int Rev Qual Res. 2025;17(4):467–92. doi: 10.1177/19408447241241501 PMC13021073 41907825

[73] Lokot M, Zreik T, El Masri R, Chaar S, Ali R, Meksassi B, et al. Power dynamics and participation within humanitarian coordination groups: A case study of the MHPSS Taskforce in Lebanon. PLOS Glob Public Health. 2024;4(3):e0003041. doi: 10.1371/journal.pgph.0003041 38483952 PMC10939209

[74] London School of Hygiene and Tropical Medicine. GOAL | LSHTM. 2025. Accessed 2026 July 17. https://www.lshtm.ac.uk/research/centres-projects-groups/goal

[75] Destrooper T. Reverse standard-setting: Indigenous women’s rights and the challenge of local concepts. Human rights and context. London: Palgrave Macmillan; 2015. 110–30.

[76] Talcott M. Together we have power: Personal traumas and political responses among activist oaxaqueñas. Lat Am Perspect. 2014;41(1):72–88.

[77] Sabelo JNG. Why decoloniality in the 21st Century?. The Thinker. 48.

[78] Mlodoch K. We want to be remembered as strong women, not as shepherds: Women Anfal survivors in Kurdistan-Iraq struggling for agency and acknowledgment. J Middle East Womens Stud. 2012;8(1):63–91. doi: 10.2979/jmiddeastwomstud.8.1.63

[79] Grant K. Mental-health support is scarce in Nunavut, but an Inuit-language counsellor training program could change that. The Globe and Mail. Accessed 2026 July 17. https://www.theglobeandmail.com/canada/article-mental-health-support-is-scarce-in-nunavut-but-an-inuit-counsellor/

[80] Rohner T. Security at Nunavut’s isolation hubs contributed to stress, tension, gov’t emails show. 2021. Accessed 2026 July 17. https://www.cbc.ca/news/canada/north/nunavut-government-security-isolation-hubs-1.6058375

[81] Patar D. Nunavut’s 14-day isolation hubs ‘offside the law and the latest science,’ says civil liberties watchdog. Nunatsiaq News. 2021.

[82] Carel H, Kidd IJ. Epistemic injustice in healthcare: A philosophial analysis. Med Health Care Philos. 2014;17(4):529–40. doi: 10.1007/s11019-014-9560-2 24740808

[83] Green G, Boaz A, Stuttaford M. Editorial: Public participation in health care: Exploring the co-production of knowledge. Front Sociol. 2019;4:73. doi: 10.3389/fsoc.2019.00073 33869395 PMC8022568

[84] Rose D, Kalathil J. Power, privilege and knowledge: The untenable promise of co-production in mental “health.”. Frontiers in Sociology. 2019;4. doi: 10.3389/fsoc.2019.00057 PMC8022626 33869380

